# Coherence Abnormalities in Tunisian Schizophrenic patients: case-control study.

**DOI:** 10.1192/j.eurpsy.2023.2282

**Published:** 2023-07-19

**Authors:** M. Mnif, L. Triki, N. Smaoui, D. Jardak, R. Feki, G. Imen, S. Omri, N. Charfi, J. Ben Thabeut, L. Zouari, M. Maalej, K. Masmoudi, M. Maalej

**Affiliations:** 1 Psychiatry C department, Hedi Chakeur Hospital; 2 Functional exploration department, Hbib Borguiba Hospital, Sfax, Tunisia

## Abstract

**Introduction:**

Resting state electroencephalogram (EEG) abnormalities in schizophrenia (SCZ) suggest alterations in neural oscillatory activity. However, few studies have examined EEG coherence in this population.

**Objectives:**

Therefore, this study investigated whether these electrophysiological characteristics differentiate schizophrenic patients from healthy controls.

**Methods:**

This was a cross-sectional, descriptive, and analytical case-control study. The selected patients were followed for SCZ at the psychiatry "C" department at the Hedi Chaker hospital of Sfax. Patients were assessed by the Positive and Negative Schizophrenia scale (PANSS) and the Treatment Adherence Scale (MARS). They all benefited from an EEG at the service of functional explorations in Sfax. Student’s test was performed to compare the coherence values between groups.

**Results:**

Thirty men including 15 schizophrenic patients and 15 age- and sex-matched controls were included. The average age was 40 years ±12.72 years for schizophrenics and 47.93 ± 15.61 years for healthy controls. Schizophrenics had an average PANSS of 64.6±22.7, and an average MARS score of 5.8 ± 3.09.

In terms of intra-hemispheric coherence, Schizophrenic patients generally exhibited higher coherence at **the Delta band** compared to healthy controls. In contrast, schizophrenic patients appeared to have decreased intra-hemispheric connectivity for other frequency bands, particularly between the frontal and other brain lobes bilaterally.

**Conclusions:**

In this study, we found that the schizophrenic patients had significantly higher coherence in the delta frequency band compared to the normal controls. These findings suggest that EEG can be a sensitive measure for diagnosing SCZ.

**Disclosure of Interest:**

None Declared

